# Supplementary material for: HIVprotI: an integrated web based platform for prediction and design of HIV proteins inhibitors
Source: J Cheminform. 2018 Mar 9;10:12. doi: 10.1186/s13321-018-0266-y (PMC5845081; doi:10.1186/s13321-018-0266-y)
Supplement: Supplementary file 2 — Additional file 2. Source code of HIVProtI web server. [file 13321_2018_266_MOESM2_ESM.zip › HIVprotI_Source-code/hivprotI/weka-3-6-6/doc/weka/classifiers/trees/m5/Rule.html]

Rule


---


|  |  |  |
| --- | --- | --- |
| **Overview** \| **Package** \| **Class** \| **Tree** \| **Deprecated** \| **Index** \| **Help** \| **Weka's home** | |  |
| **PREV CLASS**   **NEXT CLASS** | **FRAMES**    **NO FRAMES**     **All Classes** |  |
| SUMMARY: NESTED \| FIELD \| CONSTR \| METHOD | DETAIL: FIELD \| CONSTR \| METHOD |  |


---


## weka.classifiers.trees.m5 Class Rule

```
java.lang.Object
  weka.classifiers.trees.m5.Rule
```

**All Implemented Interfaces:**: java.io.Serializable, RevisionHandler

---

``` public class Rule extends java.lang.Object implements java.io.Serializable, RevisionHandler ```

Generates a single m5 tree or rule

**Version:**
:   $Revision: 6260 $

**Author:**
:   Mark Hall

**See Also:**: Serialized Form

---

**Constructor Summary**

`Rule()`
  
          Constructor declaration


| **Method Summary** | |
| --- | --- |
| `void` | `buildClassifier(Instances data)`             Generates a single rule or m5 model tree. |
| `double` | `classifyInstance(Instance instance)`             Calculates a prediction for an instance using this rule or M5 model tree |
| `void` | `freeNotCoveredInstances()`             Free up memory consumed by the set of instances not covered by this rule. |
| `RuleNode` | `getM5RootNode()` |
| `double` | `getMinNumInstances()`             Get the minimum number of instances to allow at a leaf node |
| `boolean` | `getRegressionTree()`             Get the value of regressionTree. |
| `java.lang.String` | `getRevision()`             Returns the revision string. |
| `boolean` | `getSmoothing()`             Get whether or not smoothing has been turned on |
| `boolean` | `getUnpruned()`             Get whether unpruned tree/rules are being generated |
| `boolean` | `getUseTree()`             get whether an m5 tree is being used rather than rules |
| `Instances` | `notCoveredInstances()`             Get the instances not covered by this rule |
| `void` | `setMinNumInstances(double minNum)`             Set the minumum number of instances to allow at a leaf node |
| `void` | `setRegressionTree(boolean newregressionTree)`             Set the value of regressionTree. |
| `void` | `setSmoothing(boolean s)`             Smooth predictions |
| `void` | `setUnpruned(boolean unpruned)`             Use unpruned tree/rules |
| `void` | `setUseTree(boolean u)`             Use an m5 tree rather than generate rules |
| `RuleNode` | `topOfTree()`             Returns the top of the tree. |
| `java.lang.String` | `toString()`             Return a description of the m5 tree or rule |

**Methods inherited from class java.lang.Object**

`equals, getClass, hashCode, notify, notifyAll, wait, wait, wait`

**Constructor Detail**

### Rule

```
public Rule()
```

:   Constructor declaration


**Method Detail**

### buildClassifier

```
public void buildClassifier(Instances data)
                     throws java.lang.Exception
```

:   Generates a single rule or m5 model tree.

    :   **Parameters:**: `data` - set of instances serving as training data **Throws:**: `java.lang.Exception` - if the rule has not been generated successfully

---


### classifyInstance

```
public double classifyInstance(Instance instance)
                        throws java.lang.Exception
```

:   Calculates a prediction for an instance using this rule
    or M5 model tree

    :   **Parameters:**: `instance` - the instance whos class value is to be predicted **Returns:**: the prediction **Throws:**: `java.lang.Exception` - if a prediction can't be made.

---


### topOfTree

```
public RuleNode topOfTree()
```

:   Returns the top of the tree.

---


### toString

```
public java.lang.String toString()
```

:   Return a description of the m5 tree or rule

    :   **Overrides:**: `toString` in class `java.lang.Object`
    :   **Returns:**: a description of the m5 tree or rule as a String

---


### setUnpruned

```
public void setUnpruned(boolean unpruned)
```

:   Use unpruned tree/rules

    :   **Parameters:**: `unpruned` - true if unpruned tree/rules are to be generated

---


### getUnpruned

```
public boolean getUnpruned()
```

:   Get whether unpruned tree/rules are being generated

    :   **Returns:**: true if unpruned tree/rules are to be generated

---


### setUseTree

```
public void setUseTree(boolean u)
```

:   Use an m5 tree rather than generate rules

    :   **Parameters:**: `u` - true if m5 tree is to be used

---


### getUseTree

```
public boolean getUseTree()
```

:   get whether an m5 tree is being used rather than rules

    :   **Returns:**: true if an m5 tree is being used.

---


### setSmoothing

```
public void setSmoothing(boolean s)
```

:   Smooth predictions

    :   **Parameters:**: `s` - true if smoothing is to be used

---


### getSmoothing

```
public boolean getSmoothing()
```

:   Get whether or not smoothing has been turned on

    :   **Returns:**: true if smoothing is being used

---


### notCoveredInstances

```
public Instances notCoveredInstances()
```

:   Get the instances not covered by this rule

    :   **Returns:**: the instances not covered

---


### freeNotCoveredInstances

```
public void freeNotCoveredInstances()
```

:   Free up memory consumed by the set of instances
    not covered by this rule.

---


### getRegressionTree

```
public boolean getRegressionTree()
```

:   Get the value of regressionTree.

    :   **Returns:**: Value of regressionTree.

---


### setRegressionTree

```
public void setRegressionTree(boolean newregressionTree)
```

:   Set the value of regressionTree.

    :   **Parameters:**: `newregressionTree` - Value to assign to regressionTree.

---


### setMinNumInstances

```
public void setMinNumInstances(double minNum)
```

:   Set the minumum number of instances to allow at a leaf node

    :   **Parameters:**: `minNum` - the minimum number of instances

---


### getMinNumInstances

```
public double getMinNumInstances()
```

:   Get the minimum number of instances to allow at a leaf node

    :   **Returns:**: a `double` value

---


### getM5RootNode

```
public RuleNode getM5RootNode()
```

---


### getRevision

```
public java.lang.String getRevision()
```

:   Returns the revision string.

    :   **Specified by:**: `getRevision` in interface `RevisionHandler`
    :   **Returns:**: the revision


---


|  |  |  |
| --- | --- | --- |
| **Overview** \| **Package** \| **Class** \| **Tree** \| **Deprecated** \| **Index** \| **Help** \| **Weka's home** | |  |
| **PREV CLASS**   **NEXT CLASS** | **FRAMES**    **NO FRAMES**     **All Classes** |  |
| SUMMARY: NESTED \| FIELD \| CONSTR \| METHOD | DETAIL: FIELD \| CONSTR \| METHOD |  |


---
